# Effects of Oral Supplementation with Docosahexaenoic Acid (DHA) plus Antioxidants in Pseudoexfoliative Glaucoma: A 6-Month Open-Label Randomized Trial

**DOI:** 10.1155/2018/8259371

**Published:** 2018-09-17

**Authors:** Stéphanie Romeo Villadóniga, Elena Rodríguez García, Olatz Sagastagoia Epelde, M. Dolores Álvarez Díaz, Joan Carles Domingo Pedrol

**Affiliations:** ^1^Service of Ophthalmology, Complejo Hospitalario Universitario de Ferrol, Ferrol, A Coruña, Spain; ^2^Clinical Analysis Laboratory, Complejo Hospitalario Universitario de Ferrol, Ferrol, A Coruña, Spain; ^3^Department of Biochemistry and Molecular Biomedicine, Faculty of Biology, University of Barcelona, Barcelona, Spain

## Abstract

**Purpose:**

To assess the effects of antioxidant oral supplementation based on docosahexaenoic acid (DHA) in pseudoexfoliative (PEX) glaucoma.

**Patients and Methods:**

A prospective 6-month open-label randomized controlled trial was conducted in patients with PEX glaucoma and adequate intraocular pressure (IOP) control. Patients in the DHA group received a high-rich DHA (1 g) nutraceutical formulation. Ophthalmological examination, DHA erythrocyte membrane content (% total fatty acids), plasma total antioxidant capacity (TAC), plasma malondialdehyde (MDA), and plasma IL-6 levels were assessed.

**Results:**

Forty-seven patients (DHA group 23, controls 24; mean age 70.3 years) were included. In the DHA group, the mean IOP in the right eye decreased from 14.7 [3.3] mmHg at baseline to 12.1 [1.5] mmHg at 6 months (*P*=0.01). In the left eye, IOP decreased from 15.1 [3.3] mmHg at baseline to 12.2 [2.4] mmHg at 6 months (*P*=0.007). DHA erythrocyte content increased in the DHA group, with significant differences versus controls at 3 months and 6 months (8.1% [0.9] vs. 4.4% [0.7]; *P* < 0.0001). At 6 months and in the DHA group only, TAC levels as compared with baseline increased significantly (919.7 [117.9] vs. 856.9 [180.3] *µ*M copper-reducing equivalents; *P*=0.01), and both MDA (4.4 [0.8] vs. 5.2 [1.1] nmol/mL; *P*  =  0.02) and IL-6 (2.8 [1.3] vs. 4.7 [2.3] pg/mL; *P*=0.006) levels were lower than in controls.

**Conclusions:**

Targeting pathophysiology mechanisms of PEX glaucoma by reducing oxidative stress and inflammation with a high-rich DHA supplement might be an attractive therapeutic approach. Despite the short duration of treatment, decrease in IOP supports the clinical significance of DHA supplementation.

## 1. Introduction

Pseudoexfoliative (PEX) glaucoma has been widely described as the result of the accumulation of pseudoexfoliative material, which obstructs the trabecular meshwork (TM) leading to an increase in intraocular pressure (IOP) levels. PEX glaucoma is the most common identifiable secondary form of open-angle glaucoma, accounting for up to 25% of glaucoma cases in the world [1, 2]. Compared with primary open-angle glaucoma, PEX glaucoma is associated with greater mean IOP, more advanced visual field loss at diagnosis, and poorer treatment response [3].

The etiopathogenetic mechanisms of pseudoexfoliation syndrome/PEX glaucoma are still not well understood. Decreased levels of antioxidant capacity might be involved in glaucomatous TM and neuronal damage as a result of local inadequate defense against oxidative stress [4, 5]. In the presence of reactive oxygen species (ROS), nitric oxide produces toxic metabolites (peroxynitrites) and the associated oxidative-nitrative stress induces sustained inflammation, cell proliferation, and/or neurotoxicity [5]. Oxidative DNA damage is significantly increased in the ocular epithelium regulating aqueous humor outflow in the TM. Oxidative damage constitutes an important pathogenetic step–triggering TM degeneration, which results in intraocular hypertension [6]. Also, the transcriptional factor NF-kappaB (NF-kB) can be activated by increased IOP, increased age, vascular disease, and oxidative stress [7, 8]. Overstimulation of NF-kB is also involved in the amplification of the inflammatory cascade [8]. Other studies have shown that interleukin-6 (IL-6) is linked to the pathogenesis of glaucoma [9]. Dysregulation of proinflammatory cytokines seems to be implicated in pseudoexfoliation syndrome, with high production of IL-6 at early stages of the disease inducing production of TGF-*β*_1_ and fibrotic proteins [10]. Moreover, the IL-6 family and their signal transducer glycoprotein (gp130) have been shown to be involved in inflammation and cell survival in glaucoma [11], as well as to play a specific role in the progression of retinal ganglion cell axonopathy from functional deficits to structural degeneration [12].

In recent years, there has been growing interest of the health benefits of omega-3 long-chain polyunsaturated fatty acids (*ω*-3 PUFAs), particularly docosahexaenoic acid (DHA), the pleiotropic actions of which may affect molecular pathways involved in the pathogenesis of ocular diseases, such as age-related macular edema [13], diabetic retinopathy and diabetic macular edema [14, 15], and dry eye syndrome in glaucoma patients [16]. The rationale of the use of DHA for improving retinal function is based on the inhibitory effect of DHA on the activation NF-*κ*B and synthesis of inflammatory cytokines [17, 18], generation of eicosanoids and stimulation of inflammation resolving docosanoids (resolvins and protectins) [19], antiangiogenic effects of *ω*-3 PUFAs in human endothelial cells, and antioxidant protective effects on retinal pigment epithelium and photoreceptors [20, 21]. Recently, dietary consumption of PUFAs in glaucoma has been proposed as a modifiable factor for IOP regulation through docosanoids-driven increase of aqueous outflow [22], reversal of *ω*-3 and *ω*-6 imbalance in red blood cell membranes [23, 24], and improvement of glaucomatous optic neuropathy [25]. However, the clinical experience with dietary intake of *ω*-3 PUFAs in glaucoma is very limited [26, 27].

Therefore, a midterm prospective open-label randomized controlled trial was conducted to assess the effects of oral supplementation with a nutraceutical formulation based on high-rich DHA plus vitamins and minerals in patients with PEX glaucoma. It was hypothesized that DHA supplementation improves antioxidant protection and ameliorates subclinical inflammation in PEX glaucoma.

## 2. Methods

### 2.1. Study Design

A prospective, randomized, open-label controlled study was conducted at the Service of Ophthalmology of an acute-care hospital in Ferrol, A Coruña, Spain. The duration of the study was 6 months. The objective of the study was to determine the effects of daily supplementation with a nutraceutical formulation rich in DHA plus vitamins and minerals on clinical and biochemical parameters in patients with PEX glaucoma. Ethical approval for this study was provided by the Clinical Research Ethics Committee of the autonomous community of Galicia, Spain. All participants provided written informed consent before enrollment. The study was conducted in accordance with principles of the Declaration of Helsinki and guidelines for Good Clinical Practice. The study was registered in the European Clinical Trials Database (EudraCT) (EudraCT trial number 2014-001104-21 for the Sponsor's protocol code number: GLAUPIO).

### 2.2. Participants

Between July 2016 and February 2017, all consecutive patients of both sexes aged between 18 and 70 years diagnosed with initial or moderate PEX glaucoma (stages 1 and 2 of the Hodapp-Parrish-Anderson classification) [28] were invited to participate in the study during an ophthalmologic appointment at the study center. Good control of IOP with IOP-lowering medications was an inclusion criterion. Patients unable to participate in the study according to the criteria of the ophthalmologist, pregnant women, and those who refused to sign the written consent were excluded. Patients using nutritional supplement including vitamins, minerals, fatty acids, and trace elements and those with hypersensitivity to these compounds were also excluded. Patients were specifically asked if they were already supplementing their diet with DHA (i.e., fish or flax seed oil).

### 2.3. Study Intervention

Each participant contributed 2 study eyes to the protocol. Study patients were consecutively assigned with a 1 : 1 sequential allocation to the DHA supplementation (experimental) group or to the control group using www.random.org (Randomness and Integrity Services Ltd., Dublin, Ireland). Patients assigned to the control group met all eligibility conditions, so that the selection criteria (inclusion and exclusion) were identical for all study patients. Subjects in the control group were masked regarding the existence of the experimental group. Patients in the DHA group received a high-rich DHA (1,050 mg/day) nutraceutical formulation (BrudyPio 1.5 g; Brudy Lab, S.L., Barcelona, Spain). This is a concentrated DHA triglyceride having a high antioxidant activity patented to prevent cellular oxidative damage [29, 30].  Table 1 shows the composition of the nutraceutical formulation, which includes a high dose of DHA (1 g), eicosapentaenoic acid (EPA), and a mixture of B vitamins, vitamins C, E, lutein, zeaxanthin, and minerals. All fatty acids were present in the form of triglycerides (>95%) or ethyl esters (<5%). Patients were instructed to take 3 capsules of BrudyPio 1.5 g once daily. Also, patients were told not to change glaucoma medications during the study.

### 2.4. Study Procedures

The duration of the study was 6 months. All patients were evaluated at baseline and at 3 and 6 months thereafter. At each visit, patients underwent a complete ophthalmologic examination, including slit-lamp examination, best corrected visual acuity (BCVA) (in decimals), IOP, corneal pachymetry, and retinal nerve fiber layer thickness (RNFLT) using by spectral-domain optical coherence tomography (SD-OCT) (Topcon 3D OCT-1000, de Topcon Cooperation, Tokyo, Japan). In all participants, IOP was measured by the same investigator (SRV) using a Perkins handheld applanation tonometer and during morning hours between 9:00 and 12:00 AM. Biochemical analyses included DHA erythrocyte membrane content, plasma total antioxidant capacity (TAC), plasma malondialdehyde (MDA), and plasma IL-6 levels.

At the baseline visit, eligibility criteria were checked and patients were fully informed of the purpose of the study and were requested to sign the informed consent. At the baseline visit, the nutraceutical formulation was delivered to the patient for 30-day treatment. The same assessments as in the baseline visit were performed at 1, 3, and 6 months (final visit), except for TAC, MDA, and IL-6 levels which were measured at baseline and at 6 months. At the 1-month and 3-month visits, the nutraceutical formulation was provided for the following 60 and 90 days, respectively. Compliance with DHA supplementation was assessed at the study visits by return of supplementation tablet counts and analytical data especially erythrocyte membrane DHA content. Ophthalmologists paid special care to insist on the importance of compliance with the dietary supplement and the benefit that the patient may receive from the supplement. At study visits, adverse events were recorded by questioning the patient. Patients could withdraw from the study of their own free will or be removed according to the ophthalmologist's criteria due to adverse events, concomitant diseases, or any other medical reasons.

### 2.5. Biochemical Analyses

Biochemical measurements included the composition of fatty acids on the erythrocyte membrane (*ω*-3 DHA) and levels of TAC, MDA, and IL-6 I plasma samples. The methods used for assessment of *ω*-3 DHA in the erythrocyte membrane and plasma TAC and IL-6 levels have been previously described in detail [31]. The content of *ω*-3 DHA of the erythrocyte membrane was expressed in percentages as relative amounts of total fatty acids (FA), plasma TAC as *µ*M copper-reducing equivalent values, and plasma IL-6 as pg/mL. The level of MDA was measured colorimetrically in a Synergy™ H1M, Hybrid Multi-Mode Microplate Reader (BioTek Instruments, Inc., Winooski, VT, USA). The absorbance was detected at 532 nm. MDA quantitation results are expressed as *µ*mol/L (µM) concentration using the MDA standard curve obtained during processing the plasma samples.

### 2.6. Statistical Analysis

The sample size of 27 patients per group (total 54) was calculated based on a variance of 0.048 of TAC plasma levels in PEX glaucoma, with a difference of 0.15, a power of 80%, and a type I error of 0.05. The sample size was increased to 30 patients per arm (total 30 patients) to account for a 10% loss. Categorical variables are expressed as frequencies and percentages, and continuous variables as mean and standard deviation (SD). The chi-squared (*χ*^2^) test or the Fisher's exact test was used for the analysis of categorical variables and the Student's *t*-test or the Wilcoxon signed-rank test or the Friedman test for the comparison of quantitative variables according to the conditions of application. The analysis of variance (ANOVA) for repeated measures (within subject factor: baseline, 3 and 6 months; between subject factor: experimental or control), with Bonferroni's correction was used for the comparison of study variables collected from the participants throughout the study. Changes of IOP were analyzed for the right and left eyes separately, and also as one group (both eyes together). Statistical significance was set at *P* < 0.05. Statistical analyses were performed using the Statistical Package for the Social Sciences (SPSS®) program (IBM, Armonk, New York, USA) version 21.0.

## 3. Results

During the study period, a total of 47 patients met the inclusion criteria and were included in the study, 23 of which were randomized to the experimental group and 24 to the control group. There were 25 men and 22 women, with a mean (SD) of 70.3 (5.0) years. Statistically significant differences between patients in the study groups regarding baseline characteristics were not found (Table 2). The distribution of comorbid diseases was also similar in the two study groups.

**Figure 1:**
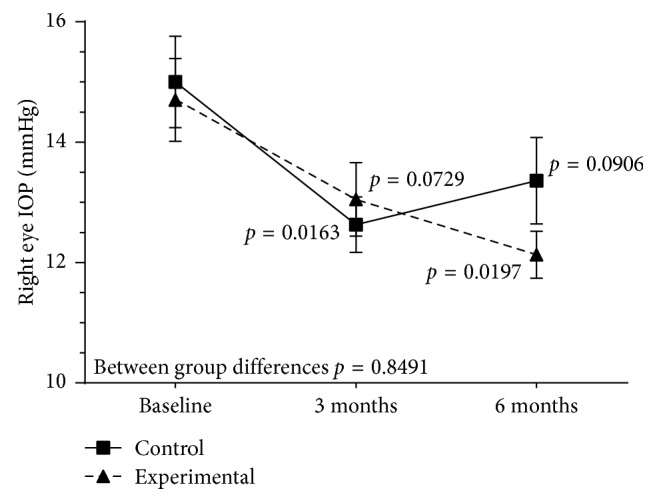
Changes of IOP values in the right eye in the experimental and control groups throughout the study.

**Figure 2:**
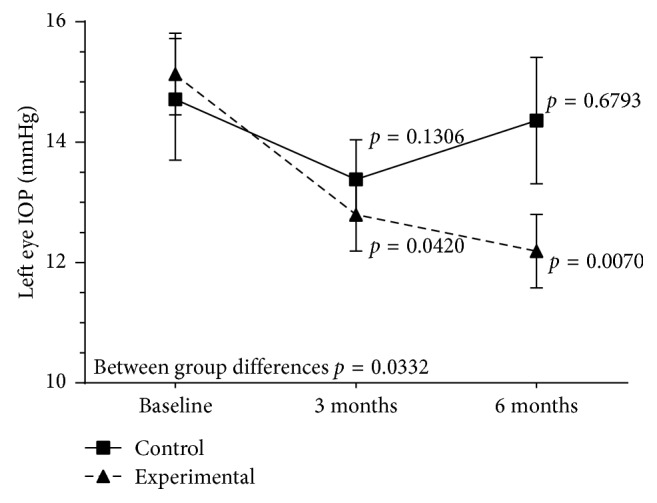
Changes in IOP values in the left eye in the experimental and control groups throughout the study.

Patients in the experimental group showed decreases of IOP from 14.7 (3.3) mmHg at baseline to 13.0 (2.7) mmHg at 3 months (*P* = 0.072) and 12.1 (1.5) mmHg at 6 months (*P* = 0.01) in the right eye. In the left eye, the mean IOP decreased from 15.1 (3.3) mmHg at baseline to 12.8 (2.6) mmHg at 3 months (*P*=0.042) and 12.2 (2.4) mmHg at 6 months (*P*=0.007). In controls, decreased IOP at 6 months as compared with baseline was not statistically significant neither in the right or left eyes. Between group differences for changes of IOP throughout the study were statistically significant (*P*=0.033) for the left eye only (Figures 1 and 2). The analysis of both eyes together showed statistically significant differences of mean IOP values between 3 and 6 months versus baseline in the experimental group, whereas in controls decreases of IOP were only significant at 3 months (Table 3). Changes in BCVA and RNFLT during the study period were not observed in any of the study groups.

**Figure 3:**
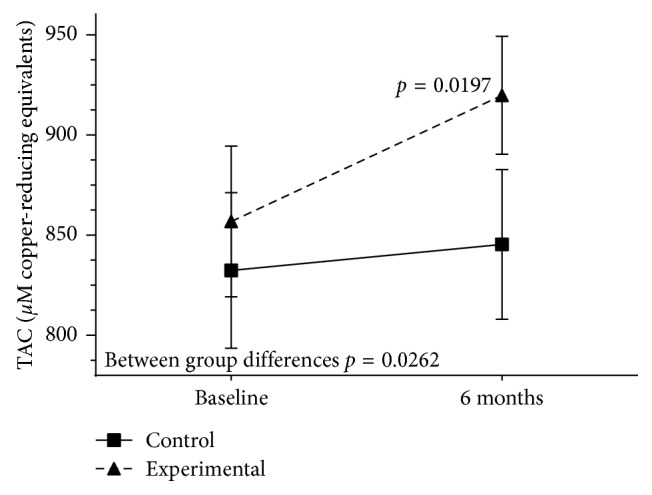
Changes of plasma total antioxidant capacity (TAC) levels in the experimental and control groups throughout the study.

The content of DHA in the erythrocyte membrane (% total fatty acids) increased in the experimental group only, with significant differences as compared with controls at 3 (7.7 [1.4] vs. 4.4 [0.7] and 6 months (8.1 [0.9] vs. 4.4 [0.7] (between group differences *P* < 0.0001). At 6 months and in the experimental group only, TAC levels as compared with baseline increased significantly (919.7 [117.9] vs. 856.9 [180.3] *µ*M copper-reducing equivalents; *P*=0.01) (between group differences *P*=0.02) (Figure 3).

**Figure 4:**
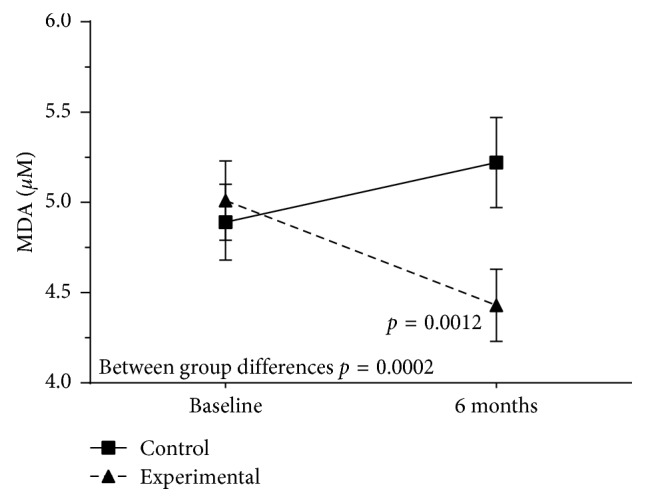
Serum malondialdehyde (MDA) values decreased significantly in the experimental group throughout the study.

**Figure 5:**
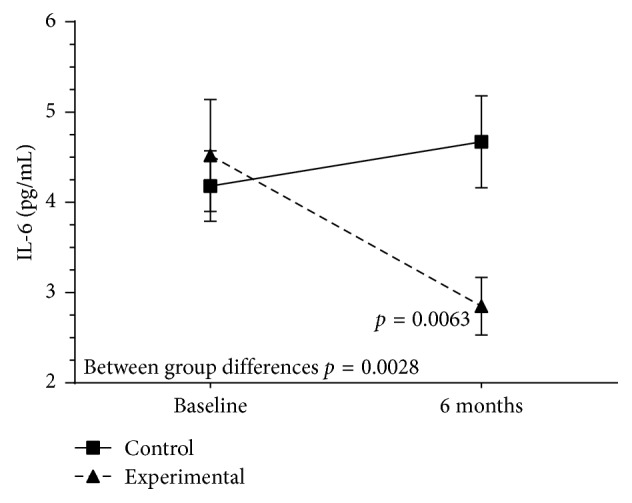
Changes of plasma interleukin- (IL-) 6 values in the experimental and control groups throughout the study.

Also, as shown in Figures 4 and 5, both MDA and IL-6 levels decreased significantly at 6 months versus baseline in the experimental group only (MDA 4.4 [0.8] vs. 5.0 (0.9) *µ*m, *P* = 0.001; and IL-6 2.8 [1.3] versus 4.7 [2.3] pg/mL, *P*=0.006). Between group differences were significant for both MDA (*P*=0.0002) and IL-6 (*P*=0.02).

The nutraceutical formulation was well tolerated, and no adverse events were registered. In relation to compliance with the nutraceutical supplement, all patients in the DHA supplementation group reported having taken the three capsules each day of the study. Also, none of the patients had their medications changed during the study.

## 4. Discussion

In the present series of patients with early or moderate PEX glaucoma, daily ingestion of a nutraceutical supplement based on high-rich DHA triglyceride plus vitamins and minerals for 6 months was associated with a trend of amelioration of IOP and clear improvement of biochemical parameters related to oxidative stress, lipid peroxidation, and inflammation. These midterm results are encouraging since a simple dietary intervention may provide an adjunct therapeutic option for patients with PEX glaucoma.

The benefits derived from omega-3 supplementation in patients with glaucoma highlight the potential targets underlying the action of DHA in response to the pathophysiological mechanisms of oxidative stress and inflammation present in open-angle glaucoma. Patients with open-angle glaucoma exhibit low levels of circulating glutathione, suggesting a general compromise of the antioxidative defense [32]. Glutathione plays a critical role in many biological processes in mammalian cells, providing a defense system for the protection of cells against reactive oxygen species. During aging, glutathione levels decline, thereby putting cells at increased risk of succumbing to oxidative stress [33]. Furthermore, glaucoma patients also show significant serum and aqueous humor increase in lipid peroxidation levels [34, 35]. Thus, oxidative stress may play a significant role during glaucoma course, initially damaging the trabecular meshwork, and then contributing to the alteration of the homeostasis in ganglionary cells, facilitating their death [36]. A significant correlation has been shown among human trabecular meshwork DNA oxidative damage, visual field damage, and IOP [37]. It has also been proved that the specific activity of superoxide dismutase demonstrates an age-dependent decline [38]. Moreover, apoptosis in glaucoma is triggered by endothelial dysregulation and dysfunction, hypoxia, and subclinical inflammation. All these conditions might contribute to accelerate the trabecular meshwork sclerosis favoring IOP increase and the apoptosis of retinal ganglion cells. Accordingly, an intervention affecting these mechanisms especially by decreasing oxidative stress and inflammation supports the rationale for the study.

However, little is known about the effect of antioxidant intake and prevention or amelioration of IOP in glaucoma. In an experimental study carried out in rats, an association between dietary omega-3 fatty acid intake and decrease in IOP caused by altered aqueous outflow was found [22]. The authors of this study suggested that dietary manipulation may provide a modifiable factor for IOP regulation. Data of clinical studies, however, are sparse. Kant et al. [39] used a food frequency questionnaire to assess the relation between the intake of a variety of antioxidants derived from food and dietary supplements in 474 glaucoma patients selected from the Nurses' Health Study and the Health Professionals Follow-up Study and followed for more than 10 years and did not observe any strong associations between antioxidant consumption and the risk of primary open-angle glaucoma. In a systematic review of 46 articles in which the effect of nutrients on open-angle glaucoma was studied, nitric oxide present in dark green leafy vegetables seemed to have a beneficial effect [27]. Interestingly, Wang et al. [26] analyzed the association between glaucoma and daily intake of PUFAs, including *ω*-3 fatty acids, in 3865 participants in the National Health and Nutrition Examination Survey 2005–2008 database who were 40 years or older and has available results of eye examinations. Increased levels of daily dietary intake of EPA (odds ratio [OR] 0.06, 95% CI 0.00–0.73) and DHA (OR 0.06, 95% CI 0.01–0.87) were associated with significantly lower odds of having glaucoma. In a previous open study of 1255 patients with glaucoma and dry eye syndrome treated for 12 weeks with the same nutritional supplement, a significant decrease in IOP values in both eyes as compared with baseline was observed [16]. These findings are consistent with a potential beneficial role of DHA supplementation in glaucoma patients. The nutraceutical product also includes minerals, vitamins, and other compounds, but the high DHA content is a remarkable characteristic of the supplement.

Finally, recent studies provided evidence of the association between systemic redox status and visual field damage in glaucoma patients, suggesting that lower systemic antioxidant capacity is associated with more severe visual field damage in glaucoma disease. Tanito et al. [40] assessed the correlation between the visual field sensitivity value and systemic levels of pro-oxidants and antioxidants by analyzing the blood biochemistry in 202 patients with open-angle glaucoma (OAG). Univariate and multivariate analyses suggested a positive correlation between mean value of visual field sensitivity and systemic levels of antioxidants, which may indicate that lower systemic antioxidant capacity is associated with more severe visual field damage in OAG. In another study, Asano et al. [41] examined the association between biological antioxidant potential (BAP), a biomarker of systemic antioxidative capacity, and glaucoma severity in 247 patients (480 eyes) with OAG and 66 healthy controls. Mixed-effect regression analysis showed that BAP was an independent contributing factor to glaucomatous damage, particularly in young males, suggesting that antioxidant therapy might be more effective in these patients. Biomarkers of systemic oxidative stress should therefore be regarded as valuable complementary information sources in glaucoma care.

Although our findings should be interpreted according to the small sample size and the limited duration of the study, further statistically significant reduction of IOP in patients assigned to the experimental group, who had otherwise good control of IOP at entry, supports the potential clinical usefulness of DHA supplementation in daily practice. More studies (longitudinal and randomized clinical trials) are needed to make the present results clinically applicable, but they may help ophthalmologists to suggest their patients an advice on high dietary intake of omega-3 fatty acids.

## Figures and Tables

**Table 1:** Composition of BrudyPio 1.5 g (Brudy Lab S.L., Barcelona, Spain) per capsule.

Composition	Per capsule	% recommended daily amount in one capsule	Per three capsules	% recommended daily amount in three capsules
Concentrated oil in ω-3 fatty acids (mg)	500		1,500	
TG-DHA 70%	350	—	1,050	—
EPA 8.5%	42.5	—	127.5	—
DPA 6%	30	—	90	—

Vitamins				
Vitamin A (retinol, *µ*g)	133.3	17	400	50
Vitamin C (ascorbic acid, mg)	26.7	33	80	100
Vitamin E (d-α-tocopherol, mg)	4	33	12	100
Vitamin B1 (thiamine, mg)	0.36	33	1.1	100
Vitamin B2 (riboflavin, mg)	0.46	33	1.4	100
Vitamin B3 (niacin equivalent, mg)	5.33	33	16	100
Vitamin B6 (pyridoxine, mg)	0.46	33	1.4	100
Vitamin B9 (folic acid, *µ*g)	66.7	33	200	100
Vitamin B12 (cobalamin, *µ*g)	0.83	33	2.5	100

Essential trace elements				
Zinc, mg	3.33	33	10	100
Copper, mg	0.33	33	1	100
Selenium, *µ*g	18.3	33	55	100
Manganese, mg	0.66	33	2	100

Other components				
Lutein, mg	3.33	—	10	—
Zeaxanthin, mg	0.33	—	1	—
Glutathione, mg	2	—	6	—
Lycopene, mg	2	—	6	—
Coenzyme Q10, mg	2	—	6	—
Anthocyanins, mg	5	—	15	—
Oleuropein, *µ*g	67	—	200	—

TG-DHA: triglyceride-bound DHA; EPA: eicosapentaenoic acid; DPA: docosapentaenoic acid. Note: The dosage is tested is three capsules per day, which corresponds to 100% of the recommended daily amounts of the included vitamins and minerals.

**Table 2:** Baseline characteristics of the study patients.

Variables	Total patients (*n*=47)	Study group		*P* value
		Experimental (*n*=23)	Control (*n*=24)	
Men/women	25/22	11/12	14/10	0.470
Age, years, mean (SD)	70.3 (5.0)	70.7 (4.5)	69.9 (5.6)	0.563

*Comorbid diseases*	—	—	—	—
Hypertension	19	10	9	—
Dyslipidemia	21	10	11	—
Diabetes mellitus	4	2	2	—
Chronic obstructive pulmonary disease	6	2	4	—
Osteoporosis	5	2	3	—
Ischemic heart disease	2	2	—	—
Depression	2	—	2	—
Arthrosis	2	1	1	—

*PEX glaucoma*	—	—	—	0.765
Right eye	14 (29.8)	7 (30.4)	7 (29.2)	—
Left eye	13 (27.6)	5 (21.7)	7 (29.2)	—
Both eyes	21 (44.7)	11 (47.8)	10 (41.7)	—
*BCVA, decimals, mean (SD)*	—	—	—	—

Right eye	0.90 (0.19)	0.91 (0.22)	0.89 (0.16)	0.735
Left eye	0.86 (0.21)	0.87 (0.19)	0.85 (0.22)	0.776

*IOP, mmHg, mean (SD)*	—	—	—	—
Right eye	14.8 (3.5)	14.7 (3.3)	15.0 (3.7)	0.769
Left eye	14.9 (4.2)	15.1 (3.3)	14.7 (4.9)	0.732

*Central corneal thickness, µm, mean (SD)*	—	—	—	—
Right eye	535.9 (38.6)	543.2 (36.6)	529.0 (40.0)	0.214
Left eye	535.5 (37.5)	540.1 (36.0)	531.1 (39.0)	0.416

*RNFLT, µm, mean (SD)*	—	—	—	—
Right eye	67.8 (22.1)	67.2 (20.5)	68.3 (23.8)	0.880
Left eye	74.5 (19.2)	71.7 (16.4)	76.7 (21.2)	0.416

PEX: pseudoexfoliative; BCVA: best corrected visual acuity; IOP: intraocular pressure; RNFLT: retinal nerve fiber layer thickness.

**Table 3:** Changes of intraocular pressure (IOP) during the study.

Study group	IOP, mmHg, mean (SD)					
	Right eye	*P* value^*∗*^	Left eye	*P* value^*∗*^	Both eyes	*P* value^*∗*^
*Experimental*						
Baseline	14.7 (3.3)	—	15.1 (3.3)		14.9 (3.3)	—
3 months	13.0 (2.7)	0.072	12.8 (2.6)	0.04	12.9 (2.6)	0.006
6 months	12.1 (1.5)	0.01	12.2 (2.4)	0.007	12.2 (2.0)	0.0003

*Control*						
Baseline	15.0 (3.7)	—	14.7 (4.9)	—	14.8 (4.3)	—
3 months	12.6 (2.2)	0.016	13.4 (3.2)	0.130	13.0 (2.8)	0.004
6 months	13.4 (3.4)	0.090	14.4 (4.9)	0.679	13.9 (4.2)	0.110

^*∗*^ *P* values versus baseline for all comparisons.

## Data Availability

The data used to support the findings of this study are available from the corresponding author upon request.
